# Multilocus microsatellite typing of *Leishmania* and clinical applications: a review

**DOI:** 10.1051/parasite/2015016

**Published:** 2015-05-05

**Authors:** Srikanth Aluru, Mallorie Hide, Gregory Michel, Anne-Laure Bañuls, Pierre Marty, Christelle Pomares

**Affiliations:** 1 Aix-Marseille Université Marseille France; 2 INSERM, U1065, Centre Méditerranéen de Médecine Moléculaire, C3M, Toxines Microbiennes dans la Relation Hôte Pathogènes 06204 Nice Cedex 3 France; 3 UMR MIVEGEC IRD 224-CNRS 5290, Universités Montpellier 1 et 2 Montpellier France; 4 Université de Nice Sophia Antipolis, Faculté de Médecine 06107 Nice Cedex 2 France; 5 Parasitologie-Mycologie, Centre Hospitalier Universitaire l’Archet CS 23079 06202 Nice Cedex 3 France

**Keywords:** *Leishmania*, Microsatellite, Genotyping, Molecular epidemiology, Multilocus microsatellite typing (MLMT)

## Abstract

Microsatellite markers have been used for *Leishmania* genetic studies worldwide, giving useful insight into leishmaniasis epidemiology. Understanding the geographic distribution, dynamics of *Leishmania* populations, and disease epidemiology improved markedly with this tool. In endemic foci, the origins of antimony-resistant strains and multidrug treatment failures were explored with multilocus microsatellite typing (MLMT). High genetic variability was detected but no association between parasite genotypes and drug resistance was established. An association between MLMT profiles and clinical disease manifestations was highlighted in only three studies and this data needs further confirmation. At the individual level, MLMT provided information on relapse and reinfection when multiple leishmaniasis episodes occurred. This information could improve knowledge of epidemiology and guide therapeutic choices for active chronic visceral leishmaniasis, the disease form in some HIV-positive patients.

## Abstract (fr)

Les marqueurs microsatellites ont été utilisés pour les études génétiques de *Leishmania* dans le monde et ont procuré un aperçu utile de l’épidémiologie des leishmanioses. La compréhension de la répartition géographique, de la dynamique des populations de *Leishmania* et de l’épidémiologie de la maladie est nettement améliorée avec cet outil. Dans les foyers d’endémie, les origines des souches résistantes à l’antimoine et des échecs de traitement avec plusieurs médicaments ont été explorées par typage microsatellite multilocus (MLMT). Une grande variabilité génétique a été détectée, mais aucune association entre les génotypes parasitaires et la résistance aux médicaments n’a été prouvée. Une association entre les profils MLMT et les manifestations cliniques de la maladie a été mise en évidence dans seulement trois études et ces données nécessitent d’être confirmées. Au niveau individuel, le MLMT fournit des informations sur la rechute et la réinfection lorsque plusieurs épisodes de leishmaniose ont eu lieu. Cette information pourrait améliorer la connaissance de l’épidémiologie et guider le choix thérapeutique pour la leishmaniose viscérale chronique active, la forme de la maladie chez certains patients VIH-positifs.

## 1. Introduction

Leishmaniases are worldwide vector-borne diseases caused by parasites of the genus *Leishmania*. These protozoan flagellates can cause different human disease forms, ranging from simple cutaneous lesions to fatal visceral disease [10]. The parasite and thus, the disease, are widely distributed geographically with approximately 0.2–0.4 million visceral leishmaniasis (VL) cases and 0.7–1.2 million cutaneous leishmaniasis (CL) cases each year [7]. The genus *Leishmania* is divided in two subgenera: *Leishmania* and *Viannia*. The subgenera are also divided in species complexes. *Leishmania* (*Leishmania*) *infantum* and *Leishmania* (*Leishmania*) *donovani* belong to the species complex *Leishmania donovani*, while *Leishmania* (*Leishmania*) *tropica* and *Leishmania* (*Leishmania*) *major* constitute two distinct species complexes. Numerous *Leishmania* species have been identified and the current classification is based on isoenzyme typing using multilocus enzyme electrophoresis (MLEE) [46]. MLEE, considered by the World Health Organization as the reference method for strain identification, separates *Leishmania* strains into groups through identification of their enzymatic patterns, so-called zymodemes. Genotypes were therefore identified indirectly, meaning that nucleotide substitution might not be detected by MLEE, leading to a low discrimination power. So, for detailed population genetics studies, it is essential to use genetic markers with high discriminatory potential. Analysis of highly variable, codominant microsatellite markers is a reliable alternative genotyping method. Microsatellites are repeated motifs of about 1–6 non-coding nucleotides found in all eukaryotic and prokaryotic genomes [28]. They are Mendelian codominant and neutral markers (not affected by natural selection) [28]. The mutation rate of microsatellites is often quoted in the range of 10^−3^–10^−4^ per locus per generation [19, 20]. The genetic variation at many microsatellite loci is characterized by high heterozygosity and the presence of multiple alleles which makes microsatellite sequence modifications particularly useful for studying differences between closely related organisms [20, 57]. Consequently, the analysis of microsatellite sequence variation is an important tool for population genetic studies for many species [20, 56, 57]. Moreover, multilocus microsatellite typing (MLMT) yields consistently reproducible results that are potentially exchangeable among laboratories [57]. MLMT has been used to identify, discriminate, and characterize geographically distributed populations of strains of *Leishmania* even at the intra-zymodeme level [14, 44, 57]. This review aims (i) to draw an inventory of the existing microsatellite markers, (ii) to explore the association between MLMT genotypes and clinical variations on the one hand, and drug-resistant strains in endemic foci on the other, and (iii) to discuss the use of this tool in case of several leishmaniasis episodes in patients.

## 2. MLMT as a genetic method for the genus *Leishmania*

The *Leishmania* genome is rich in microsatellite sequences with around 600 per haploid genome [47, 57]. Its structural organization reveals that the number of (CA)*n* loci has a value similar to those found in the genomes of other eukaryotes [47, 57]. In 1994, Rossi et al. [47] performed the first MLMT study on *Leishmania donovani* complex. Since this first study, many other microsatellite markers have been designed for different species of *Leishmania*. All the markers designed to date are listed in Table 1. Because the regions flanking the repeat sequences are not strongly conserved between *Leishmania* species, markers for the subgenera *Leishmania* and *Viannia* are distinct. Also, some species-specific markers have been developed (Table 1) [57]. Within the subgenus *Leishmania*, markers could be used independently in the species complex *L. donovani* (*L.* (*L.*) *donovani* and *L.* (*L.*) *infantum*). Seven of them (LIST7010, LIST7011, LIST7028, LIST7030, LIST7033, LIST7035, and LIST7036) have also been used for *L.* (*L.*) *major* and *L.* (*L.*) *tropica* [5, 14, 21, 25–27, 38, 44, 47, 48, 58]. However, for these last two species, species-specific markers have also been designed [5, 58]. All markers designed within the subgenus *Viannia* could be used for all species within this subgenus [45, 53, 54]. *L.* (*L.*) *donovani* and *L.* (*L.*) *infantum* are the species with the largest number of markers designed and the first 14 microsatellite markers (Table 1) are the most used for this species complex [14, 27, 38, 44]. Some markers have not been used since they appeared in the first study either because they are new or because they are not well documented or these markers were not polymorphic enough – for example in the case of MON-1 discrimination [25, 26, 44, 58]. Fewer markers have been developed for the subgenus *Viannia*, with 86 versus 25 for the subgenera *Leishmania* and *Viannia*, respectively (Table 1). The distinct markers show different degrees of variability. The range of the number of alleles highlights that some markers give information on a given population whereas they are not informative for another population of strains (Table 1).

**Table 1.** *Leishmania* Microsatellite markers. Classification has been made according to the number of citations in the literature. The number of alleles is listed under the species and represents the range found in the different studies. Whereas some markers have been used only once, others have been used several times and the number of strains is different from one study to another. These two points can explain the differences in allele numbers between the loci. The number of alleles is thus subject to change with other studies.

	Subgenus *Leishmania*		
Markers	*L.* (*L.*) *donovani/L.* (*L.*) *infantum*		References
† **Lm4TA**	**33–11**		[14]
† **LIST7039** **t**	**1–14**		[27]
† **Li71-33**	**1–15**		[45]
† **Lm2TG**	**1–18**		[14]
† **Li22-35**	**1–18**		[45]
† **Li45-24**	**1–13**		
† **TubCA**	**1–9**		
† **Li71-7**	**2–8**		
† **Li23-41**	**1–23**		
† **Li71-5/2**	**1–4**		
† **LIST7031**	**1–11**		[27]
‡ **Li46-67**	**1–4**		[45]
† **Li41-56**	**2–9**		
‡ **CS20**	**1–12**		[36]
‡LIST7028^mt^	2**–**3		[27]
†LIST7033^mt^	4**–**6		
‡LIST7027^t^	3**–**4		
‡LIST7029	1**–**4		
ITS1	*		[21]
‡LIST7030^mt^	1**–**3		[27]
†LIST7035^mt^	3**–**4		
†LIST7037^t^	4**–**5		
‡LIST7040^t^	1**–**4		
†LIST7021	3**–**4		
‡LIST7023	1**–**3		
‡LIST7024	3**–**7		
†LIST7025	3**–**8		
†ISA136	3		[48]
‡ST436	4		
LIST7010^mt^	3		[26]
LIST7011^mt^	2**–**3		
‡LIST7036^mt^	4		[27]
‡LIST7022	2**–**3		
†LIST7026	2**–**6		
†LIST7032	2**–**3		
‡LIST7034	3**–**5		
†LIST7038	3		
CS19	4**–**8		[36]
‡Li71-19	1		[45]
‡Li72-14	1		
†Li72-20	4		
‡DPB1	3**–**4		[50]
‡DPB2	3**–**6		
‡HG	3**–**6		
‡Rossi1	2**–**5		
†Rossi2	5		
LIST7001^m^	2		[26]
LIST7002^m^	1		
LIST7003^m^	2		
LIST7004^m^	1		
LIST7005^m^	2		
LIST7006^m^	1		
LIST7007^m^	1		
LIST7008^m^	2		
LIST7009^m^	2		
LIST7012^m^	2		
LIST7013^m^	2		
†LiBTG	8		[25]
†LiBTA	7		
‡Li21-34	Uk		[45]
‡Li71-42	Uk		
‡Li72-17/2	Uk		
Markers	*L.* (*L.*) *major*		References
LIST7028	3		[27]
LIST7033	4		
LIST7030	3		
LIST7035	4		
LIST7036	4		[26]
LIST7010	4		
LIST7011	2–5		[58]
4GTG^t^	2–3		
27GTG^t^	3		[5]
36GTG	3–4		
39GTG	2–5		
45GTG	5–7		
IGC	2		
28AT	5–6		
7IAT	3–9		
IGACA	1–3		
ICA	4–7		
LIST7001	4		[26]
LIST7002	5		
LIST7003	3		
LIST7004	4		
LIST7005	4		
LIST7006	4		
LIST7007	4		
LIST7008	2		
LIST7009	5		
LIST7012	3		
LIST7013	3		
Markers	*L.* (*L.*) *tropica*		References
LIST7039	5–15		[27]
LIST7028	3		
LIST7033	4–5		
LIST7027	4–10		
LIST7030	3		
LIST7035	4		
LIST7036	4–7		
LIST7037	4		
LIST7040	4–7		
LIST7010	3–9		[26]
LIST7011	2–8		
GA1	2–3		[58]
GA2	6–8		
GA3	3–8		
GA6	3		
GA9	2–4		
GA10	3–5		
GA11	4		
Mix9	2		
GM2	3–4		
GTG1	3		
GTG3	3–5		
GT4	4–6		
GACA4	2		
GACA1	3		
4GTG	3		
27GTG	4		
Subgenus *Viannia*			
Markers	*L.* (*V.*) *braziliensis*	*L.* (*V.*) *guyanensis*	References
AC01/AC01R	4–16	4–10	[55]
AC16/AC16R	8–14	2–5	
AC52	19–22	4–10	
ITSbraz	6	2–5	[54]
LRC	12–15	5–8	
EMI	12–14	7–9	
GO9	7–10	4–5	
E11	6–9	3–7	
ARP	15–18	7–8	
Ibh3	5–9	3–8	
CAK	8–13	5–6	
LBA	8–14	3–7	
CSg46	9–14	4–5	[46]
CSg47	14–29	11	
CSg53	3–13	2–4	
CSg55	2–13	6–12	
CSg59	3–7	3	
7GN	10–17	3–5	
11H	4–17	6–8	
11C	6–17	3–4	
6F	8–16	3–8	
10F	4–9	2	
B6F	12–16	3–4	
B3H	10–14	4–5	
CSg48	4–19	2–4	

The 14 first microsatellite markers (in bold) are the most used in *L.* (*L.*) *donovani/L.* (*L.*) *infantum* studies. Markers used for several species are tagged with ^t^ and/or ^m^ for *L.* (*L.*) *tropica* and/or *L.* (*L.*) *major*, respectively.

* The region ITS1 contains several microsatellites and polymorphisms can only be detected by sequencing.

† mean polymorphism obtained within zymodeme MON-1.

‡ mean polymorphism obtained among zymodemes.

Thirty microsatellite markers have been developed by Kebede et al. [31] for *L.* (*L.*) *aethiopica*. Uk: Unknown.

## 3. Potential for biogeographic inference

MLMT gives important insights into the epidemiology of leishmaniases and allows characterization of strains from different geographical areas. It has been used to differentiate strains from closely related endemic areas within a region, from one country to another, and across continents [4, 5, 18, 24, 40, 42, 45, 59]. MLMT can also be used to follow the spread of parasites or to determine the origin of a specific infection (e.g. in the case of infected travelers) [5, 18, 45, 59]. Studies show, in most of cases, good correlation between the geographical origin of the strain and MLMT profiles [4, 5, 18, 24, 40, 42, 45, 59]. MLMT has been used to compare genetic profiles of *L.* (*L.*) *chagasi* and *L.* (*L.*) *infantum* strains from most endemic regions worldwide and confirmed that *L.* (*L.*) *chagasi* is in fact an *L.* (*L.*) *infantum* subpopulation imported from Southern Europe. Indeed, *L.* (*L.*) *infantum* was most probably introduced to the Americas by infected dogs with Conquistadores in the XVIth century [33, 39]. Thereby, MLMT contributes to a better understanding of the geographical distribution and dynamics of *Leishmania* populations, and disease epidemiology [4, 5, 18, 24, 33, 39, 40, 42, 45, 59].

## 4. Is it possible to correlate clinical manifestations and treatment failure of leishmaniasis through microsatellites?

MLMT is a tool to discriminate and characterize closely related strains and to determine the reproductive strategies of the genus *Leishmania* [48, 51, 52, 57]. Analyses of microsatellite sequences have provided some answers to epidemiological or biological questions concerning the population dynamics of the parasites in specific endemic foci, or the genetic exchanges between strains, and their evolution [24, 52, 57]. Microsatellites are neutral markers in non-coding regions of the genome. Therefore, clinical manifestations should not be directly linked to MLMT profiles [19, 20]. However, even though several studies have clearly demonstrated that this parasite has the capacity to genetically recombine by allogamy (interspecific recombination in the case of *Leishmania*), it displays a mainly clonal and/or endogamic (intraspecific recombination) mode of reproduction, generating strong linkage disequilibrium in the genome [35, 49, 52]. As a consequence, some phenotypes, and, thus some clinical manifestations might be associated with particular genotypes [49, 52]. For this reason, some studies have tried to find an association between MLMT profiles and clinical manifestations of the disease. The leishmaniases exhibit a wide variety of clinical symptoms ranging from asymptomatic carriage to highly complex pathological forms with cutaneous, mucocutaneous, or visceral disseminated diseases. The form of the disease is determined mainly by the parasite, the reservoir, host genetics, and the vectors [10, 41, 55]. Most studies on *L.* (*L.*) *donovani*, *L.* (*L.*) *infantum*, *L.* (*L.*) *tropica*, and the *Viannia* subgenus did not find any association between clinical manifestations (VL, CL, and PKDL (post-kala-azar dermal leishmaniasis)) and MLMT profiles [8, 23, 33–36, 44, 60, 61]. Nevertheless, three studies highlighted associations between some particular genotypes and the clinical manifestations [16, 25, 32]. Chargui et al. [16] and Hide et al. [25] found a clear association between clinical manifestations and parasite genotypes in *L.* (*L.*) *infantum* strains. Indeed, two distinct populations of strains were found with parasites isolated from CL and VL patients [16]. Similarly, a genetic difference was observed between *L.* (*L.*) *infantum* strains isolated from asymptomatic carriers and HIV-positive patients in the South of France [25]. A recent study of *L.* (*L.*) *tropica* highlighted genetic differences between the dermotropic and viscerotropic strains in India [32]. The strains of *L.* (*L.*) *tropica* isolated from human cases of CL fell into the same subpopulations of strains from human cases of VL but they were not genetically identical [32]. However, these three studies compared a small number of strains [16, 25, 32]. Further studies with more strains are needed to confirm these associations. To date, strain characterization by MLMT highlights that a large spectrum of clinical outcomes can be obtained from related strains and, in general, clinical manifestations in infected patients cannot be predicted with MLMT profiles. Indeed, the broad spectrum of clinical manifestations depends on more complex host-parasite interactions such as the parasite genotype, host susceptibility, and genetic background.

For treatment of leishmaniases, drugs currently available include: pentavalent antimony, pentamidine, various amphotericin B formulations, miltefosine, and paromomycin [2, 17]. In some countries, treatment choice is frequently associated with economic considerations and pentavalent antimonial compounds are frequently the first choice [17]. In India, antimony is the first-line drug to treat VL caused by *L.* (*L.*) *donovani* and resistance has emerged leading to treatment failure [11, 15, 63]. Drug resistance has become a major issue to control leishmaniases in some countries. The mechanisms by which drug resistance emerges and spreads are not completely known [30, 64]. MLMT has been used to study strains from patients with treatment failure in order to analyze whether a specific genotype is associated with treatment failure. Studies have been set up with strains of *L.* (*L.*) *donovani* and *L.* (*V.*) *braziliensis* resistant to pentavalent antimony [1, 38, 63]. No association was found between the genotypes and drug susceptibility or clinical outcome. Two hypotheses were proposed to explain treatment failure: a pleomorphic adaptive response to drug pressure or genetic recombination events [1, 38, 63]. Therefore, MLMT cannot be used to predict drug resistance of strains isolated from patients and other markers should be used instead [30].

## 5. Does MLMT allow study of relapse and reinfection?

In the Mediterranean basin and South America, *L.* (*L.*) *infantum* is responsible for VL, more sporadically CL, and is often an opportunistic infection in acquired immunodeficiency syndrome (AIDS) patients [6, 37]. In endemic areas of Sub-Saharan Africa and the Indian subcontinent, the co-infection rate of VL-HIV has been steadily increasing [29]. In these areas, *Leishmania* relapses and reinfections are a major concern for AIDS patients who are at a higher risk for opportunistic infections [6, 13]. Thus, multiple clinical VL episodes in AIDS patients are observed, defining active chronic visceral leishmaniasis [13]. In endemic areas, reinfection rates might be higher than estimated which may make it difficult to decide on treatment: Is the new episode of infection a relapse related to treatment failure, or has the patient acquired a new infection [43]? The enzymatic method is not sufficiently discriminant to differentiate strains responsible for relapse and strains from newly acquired infections [43]. In contrast, the high level of resolution of MLMT allows characterization of several strains isolated from patients at different clinical episodes, allowing discrimination between relapse and reinfection [12, 23, 34, 44, 60]. Knowing the MLMT profiles of *Leishmania* strains during multiple VL episodes is highly informative to follow the evolution of infection within a single individual, with the limitation that reinfection can only be detected if strains from successive episodes have different genotypes [12, 62].

## 6. Conclusion

MLMT is a highly discriminatory and reproducible tool that has been used for *Leishmania* population genetic studies worldwide [57]. All *Leishmania* species could be studied with different loci and theoretically data could be easily exchangeable among laboratories [14, 44, 47, 57]. Most studies did not find an association between MLMT profiles and clinical manifestations, except three studies on *L.* (*L.*) *infantum* and *L.* (*L.*) *tropica* species [16, 25, 32]. These contradictory results can be explained by the diversity of reproductive systems in the *Leishmania* populations (endogamy, clonality, and allogamy) and by host response that can also be diverse and consequently produce a variety of symptoms not associated with the parasite genotype. Thus, the association with clinical outcome of the disease will be population- and study-specific.

In endemic foci, no link was observed between parasite genotypes and antimony drug resistance using MLMT [1, 9, 38, 63]. Usually, *Leishmania* resistance to antimonials is assessed *in vitro* by exposing infected macrophages to various concentrations of the drug [22]. This technique is currently still the reference method even though it is time-consuming and not completely standardized [22].

At the individual level, and especially in HIV/AIDS patients who experience active chronic VL, MLMT provides information on relapse or reinfection when multiple clinical episodes occur [13, 60]. An easy-to-use method for genotyping *Leishmania* strains would be to perform a single multiplex PCR assay with at least 10 microsatellite markers [3]. This multiplex PCR will be less time-consuming and will allow more laboratories to perform such a test. In case of multiple episodes, each strain isolated could be compared to another and the treatment could be adapted according to the genotype retrieved. Identical genotypes for two episodes suggest a relapse and consequently drug resistance. In this event, physicians could adjust treatment in real time. Moreover, genetic characterization of strains by MLMT could increment a database that would give additional information as MLEE. This review highlights that MLMT should replace MLEE for epidemiological and population dynamics studies, until whole-genome sequencing takes over.
